# Supplementary material for: Unmet patient needs and information gaps in fertility counseling for persons living with HIV/AIDS: Evidence from Ghana
Source: PLOS Glob Public Health. 2026 Apr 16;6(4):e0006017. doi: 10.1371/journal.pgph.0006017 (PMC13086305; doi:10.1371/journal.pgph.0006017)
Supplement: S1 File — Consolidated criteria for reporting qualitative research (COREQ) 32-item checklist showing compliance with reporting guidelines. (DOCX) [file pgph.0006017.s001.docx]

**Supporting Information S1**

**COREQ (Consolidated Criteria for Reporting Qualitative Research) Checklist**

**Manuscript Title:** UNMET PATIENT NEEDS AND INFORMATION GAPS IN FERTILITY COUNSELING FOR PERSONS LIVING WITH HIV/AIDS: EVIDENCE FROM GHANA

**Manuscript ID:** PGPH-D-25-04006

**Authors:** Priscilla Asantewaa Boadi, Victor Luckyboy Dzramado

**Source:** Tong A, Sainsbury P, Craig J. Consolidated criteria for reporting qualitative research (COREQ): a 32-item checklist for interviews and focus groups. International Journal for Quality in Health Care. 2007;19(6):349-357.

**Instructions:** This checklist should be completed in conjunction with the manuscript. For each item, provide the page number(s) where the information is reported in the manuscript. If an item is not applicable or not reported, indicate "Not applicable" or "Not reported" with a brief explanation.

**Domain 1: Research Team and Reflexivity**

**Personal Characteristics**

| **No.** | **Item** | **Guide Questions/Description** | **Page No.** | **Description in Manuscript** |
| --- | --- | --- | --- | --- |
| 1 | **Interviewer/facilitator** | Which author/s conducted the interview or focus group? | 7, 10, 28 | The primary researcher conducting interviews was a trained female research assistant. "The primary researcher conducting interviews was a female research assistant with a Master's degree in Nursing and five years of experience in qualitative health research" (Page 7). PAB (Priscilla Asantewaa Boadi) conducted data collection (Page 28, Authors' Contributions). |
| 2 | **Credentials** | What were the researcher's credentials? E.g. PhD, MD | 7 | "The primary researcher conducting interviews was a female research assistant with a Master's degree in Nursing" (Page 7). |
| 3 | **Occupation** | What was their occupation at the time of the study? | 7 | "The primary researcher conducting interviews was a female research assistant with a Master's degree in Nursing and five years of experience in qualitative health research, particularly in HIV care and reproductive health" (Page 7). |
| 4 | **Gender** | Was the researcher male or female? | 7 | "The primary researcher conducting interviews was a female research assistant" (Page 7). |
| 5 | **Experience and training** | What experience or training did the researcher have? | 7 | "...five years of experience in qualitative health research, particularly in HIV care and reproductive health. The researcher underwent training in qualitative interviewing techniques, including development of rapport, active listening, use of probes, and maintenance of neutrality during interviews" (Page 7). |

**Relationship with Participants**

| **No.** | **Item** | **Guide Questions/Description** | **Page No.** | **Description in Manuscript** |
| --- | --- | --- | --- | --- |
| 6 | **Relationship established** | Was a relationship established prior to study commencement? | 7 | "She had no prior clinical or personal relationship with study participants, minimizing potential influence of pre-existing relationships on data collection" (Page 7). |
| 7 | **Participant knowledge of the interviewer** | What did the participants know about the researcher? E.g. personal goals, reasons for doing the research | 13-14 | Participants were informed during the informed consent process about the study's purpose and the researcher's role. "All participants provided voluntary written informed consent after receiving comprehensive information about the study's purpose, procedures, risks, benefits, and their rights as participants" (Pages 13-14). |
| 8 | **Interviewer characteristics** | What characteristics were reported about the interviewer/facilitator? E.g. Bias, assumptions, reasons for interest in the research topic | 7 | "Reflexivity, the process of examining how the researcher's background, assumptions, and positioning influence the research process, was maintained throughout data collection and analysis. The researcher maintained a reflexive journal documenting personal reactions, observations, assumptions, and methodological decisions throughout the research process" (Page 7). |

**Domain 2: Study Design**

**Theoretical Framework**

| **No.** | **Item** | **Guide Questions/Description** | **Page No.** | **Description in Manuscript** |
| --- | --- | --- | --- | --- |
| 9 | **Methodological orientation and Theory** | What methodological orientation was stated to underpin the study? E.g. grounded theory, discourse analysis, ethnography, phenomenology, content analysis | 6 | "This qualitative phenomenological study was designed to explore the lived experiences of PLWHA regarding fertility counseling and information provision at a district-level HIV care facility. The phenomenological approach was selected for its capacity to capture rich, detailed accounts of participants' experiences, perceptions, and the meanings they attribute to fertility counseling encounters within HIV care settings" (Page 6). |

**Participant Selection**

| **No.** | **Item** | **Guide Questions/Description** | **Page No.** | **Description in Manuscript** |
| --- | --- | --- | --- | --- |
| 10 | **Sampling** | How were participants selected? E.g. purposive, convenience, consecutive, snowball | 7 | "Purposive sampling was employed to select information-rich participants who could provide detailed accounts of their experiences with fertility counseling and information provision. Purposive sampling enabled deliberate selection of participants based on predetermined criteria relevant to the research objectives, ensuring inclusion of individuals with direct experience of the phenomenon under investigation" (Page 7). |
| 11 | **Method of approach** | How were participants approached? E.g. face-to-face, telephone, mail, email | 10 | Participants were approached at the ART clinic. "In-depth semi-structured interviews were conducted in private consultation rooms at St. Michael's Hospital" (Page 10), suggesting face-to-face recruitment at the clinic setting. |
| 12 | **Sample size** | How many participants were in the study? | 2, 8-9, 14 | "12 PLWHA aged 25 to 45 years" (Page 2, Abstract). "Data saturation was achieved after interviewing 12 participants" (Page 9). "Twelve PLWHA participated in this study" (Page 14). |
| 13 | **Non-participation** | How many people refused to participate or dropped out? Reasons? | Not explicitly reported | The manuscript states "No participant withdrew after providing initial consent" (Page 14), but does not report the number of people who refused to participate initially. |

**Setting**

| **No.** | **Item** | **Guide Questions/Description** | **Page No.** | **Description in Manuscript** |
| --- | --- | --- | --- | --- |
| 14 | **Setting of data collection** | Where was the data collected? E.g. home, clinic, workplace | 6, 10 | "The study was conducted at St. Michael's Hospital ART clinic located in Pramso, Ashanti Region, Ghana" (Page 6). "In-depth semi-structured interviews were conducted in private consultation rooms at St. Michael's Hospital" (Page 10). |
| 15 | **Presence of non-participants** | Was anyone else present besides the participants and researchers? | 10 | "Interviews were conducted in private settings to ensure participants could speak freely without fear of being overheard" (Page 14), suggesting no non-participants were present. Not explicitly stated in data collection section. |

**Data Collection**

| **No.** | **Item** | **Guide Questions/Description** | **Page No.** | **Description in Manuscript** |
| --- | --- | --- | --- | --- |
| 16 | **Description of sample** | What are the important characteristics of the sample? E.g. demographic data, date | 14-15, Table 1 | Comprehensive demographic characteristics provided in Table 1 (Page 15): age distribution, sex, marital status, educational level, employment status, number of living children, duration on ART. "The mean age of participants was 34.5 years (SD = 5.8). Most participants were married or cohabiting (n=8, 66.7%) and had attained at least secondary education (n=10, 83.3%)" (Pages 14-15). |
| 17 | **Interview guide** | Were questions, prompts, guides provided by the authors? Was it pilot tested? | 9-10 | "A semi-structured interview guide was developed based on extensive literature review, theoretical frameworks, and consultation with reproductive health and HIV care experts. The guide covered three primary domains... The interview guide was piloted with two PLWHA not included in the final sample. Pilot interviews enabled refinement of question wording, sequencing, and probing strategies" (Pages 9-10). |
| 18 | **Repeat interviews** | Were repeat interviews carried out? If yes, how many? | Not applicable | Not reported. Single interviews were conducted with each participant. |
| 19 | **Audio/visual recording** | Did the research use audio or visual recording to collect the data? | 10 | "All interviews were audio-recorded with participants' explicit permission to ensure accurate data capture and enable thorough analysis" (Page 10). |
| 20 | **Field notes** | Were field notes made during and/or after the interview or focus group? | 10-11 | "Detailed field notes were maintained throughout the data collection process. Field notes documented contextual observations, non-verbal cues (such as facial expressions, body language, and emotional responses), interview dynamics, environmental conditions, and researcher reflections. Field notes were reviewed immediately following each interview and integrated into the analytical process during transcript coding" (Pages 10-11). |
| 21 | **Duration** | What was the duration of the interviews or focus group? | 10 | "Interviews ranged from 45 to 90 minutes in duration, with most lasting approximately 60 minutes, sufficient to explore experiences in depth while avoiding participant fatigue" (Page 10). |
| 22 | **Data saturation** | Was data saturation discussed? | 8-9 | Comprehensive discussion of data saturation: "Data saturation was achieved after interviewing 12 participants, as evidenced by thematic redundancy across the final three interviews. At this point, no new concepts, themes, or variations emerged, and participants' accounts consistently reinforced patterns already identified in earlier interviews" (Page 9). |
| 23 | **Transcripts returned** | Were transcripts returned to participants for comment and/or correction? | Not reported | Not explicitly reported in the manuscript. |
| 24 | **Member checking** | Was member checking conducted? | 12 | "Member checking was conducted by sharing preliminary findings with a subset of participants to verify that interpretations accurately reflected their experiences. Participants confirmed that the themes resonated with their experiences and provided additional clarifications that enriched final interpretations" (Page 12). |

**Domain 3: Analysis and Findings**

**Data Analysis**

| **No.** | **Item** | **Guide Questions/Description** | **Page No.** | **Description in Manuscript** |
| --- | --- | --- | --- | --- |
| 25 | **Number of data coders** | How many data coders coded the data? | 12 | "Investigator triangulation was implemented, with two researchers independently coding a subset of transcripts and then comparing their coding to identify areas of consensus and divergence" (Page 12). Two coders: PAB and VLD. |
| 26 | **Description of the coding tree** | Did authors provide a description of the coding tree? | 11-12 | "The coding process followed a systematic multi-stage approach. First, open coding was conducted whereby transcripts were read line-by-line and initial codes were assigned to segments of text representing distinct ideas, experiences, or meanings. Following open coding, axial coding was performed to identify relationships among codes, grouping related codes into broader categories representing common concepts or experiences. Finally, selective coding integrated categories into coherent themes representing overarching patterns in the data" (Pages 11-12). While a visual coding tree is not provided, the coding process and theme development are described. |
| 27 | **Derivation of themes** | Were themes identified in advance or derived from the data? | 11 | "Data analysis employed an iterative, inductive approach consistent with phenomenological methodology" (Page 11). "Open coding remained close to the data, using participants' own language where possible to preserve the authenticity of their expressions" (Page 11). Themes were derived from the data, not pre-determined. |
| 28 | **Software** | What software, if applicable, was used to manage the data? | 11 | "NVivo qualitative data analysis software (version 12) was employed to facilitate systematic data organization, coding, and analysis" (Page 11). |

**Reporting**

| **No.** | **Item** | **Guide Questions/Description** | **Page No.** | **Description in Manuscript** |
| --- | --- | --- | --- | --- |
| 29 | **Participant quotations presented** | Were participant quotations presented to illustrate the themes/findings? Was each quotation identified? E.g. participant number | 15-20 | Extensive participant quotations presented throughout Results section with participant identification codes. Examples: "Participant 03, female, age 32" (Page 16); "Participant 07, male, age 38" (Page 16); "Participant 09, female, age 29" (Page 16), etc. Each quotation includes participant number, sex, and age. |
| 30 | **Data and findings consistent** | Was there consistency between the data presented and the findings? | 15-20 | Yes. Each sub-theme is supported by multiple participant quotations, quantitative prevalence data from tables, and field note observations that consistently illustrate the findings. The three sub-themes directly address the study objectives and are well-supported by the data presented. |
| 31 | **Clarity of major themes** | Were major themes clearly presented in the findings? | 2, 15-20 | Three major sub-themes are clearly presented: (1) Limited proactive counseling on conception safety; (2) Inadequate information on contraception-ART integration; (3) Complete absence of counseling on assisted reproductive options. Each sub-theme has a dedicated section with supporting data, tables, quotations, and field notes (Pages 15-20). Abstract also clearly states these three themes (Page 2). |
| 32 | **Clarity of minor themes** | Is there a description of diverse cases or discussion of minor themes? | 15-20 | Within each major sub-theme, the manuscript presents variations in participant experiences, including: the few participants who received some counseling (e.g., "The few participants who did receive some conception-related counseling..." Page 16); differences between serodiscordant and seroconcordant couples; variations by gender; and individual circumstances. Field notes also capture diverse emotional reactions and contextual variations. |

**Summary Statement**

This qualitative phenomenological study has been reported in accordance with the COREQ (Consolidated criteria for REporting Qualitative research) guidelines. The completed checklist demonstrates comprehensive reporting across all three domains:

**Domain 1 (Research team and reflexivity):** All 8 items addressed, including detailed researcher characteristics, credentials, experience, and reflexivity practices. The absence of prior relationships with participants and maintenance of reflexive journaling are clearly documented.

**Domain 2 (Study design):** All 16 items addressed or explained. The study clearly describes the phenomenological approach, purposive sampling strategy, setting, comprehensive data collection procedures including pilot testing, audio recording, field notes, data saturation, and member checking.

**Domain 3 (Analysis and findings):** All 8 items addressed. The manuscript provides detailed description of the multi-stage coding process, use of NVivo software, investigator triangulation, derivation of themes through inductive analysis, and clear presentation of findings with extensive participant quotations properly identified.

**Items not fully reported:**

- Item 13 (Non-participation rates and reasons): While the manuscript reports no withdrawals after consent, initial refusal rates are not documented.
- Item 15 (Presence of non-participants): While private settings are described, explicit statement about absence of non-participants during interviews is not made.
- Item 18 (Repeat interviews): Not applicable as single interviews were conducted.
- Item 23 (Transcripts returned): Not reported whether transcripts were returned to participants.

**Justification for unreported items:** Items 13 and 15 represent minor omissions that do not compromise the overall rigor and transparency of the research. Item 18 is not applicable to this study design. Item 23, while not explicitly reported, is addressed through member checking of findings (Item 24).

Overall, this study demonstrates strong adherence to COREQ guidelines with comprehensive and transparent reporting of qualitative research methods, procedures, and findings.

**Checklist Completed by:** Victor Luckyboy Dzramado

**Date:** November 25, 2025

**Declaration:** We confirm that this completed COREQ checklist accurately reflects the reporting of qualitative research methods and findings in the manuscript titled "Unmet Patient Needs and Information Gaps in Fertility Counseling for Persons Living with HIV/AIDS: Evidence from Ghana" (Manuscript ID: PGPH-D-25-04006).
