# Supplementary material for: Unmet patient needs and information gaps in fertility counseling for persons living with HIV/AIDS: Evidence from Ghana
Source: PLOS Glob Public Health. 2026 Apr 16;6(4):e0006017. doi: 10.1371/journal.pgph.0006017 (PMC13086305; doi:10.1371/journal.pgph.0006017)
Supplement: S1 Text — (DOCX) [file pgph.0006017.s002.docx]

**SUPPORTING INFORMATION**

**DE-IDENTIFIED EXCERPTS AND CODED THEMATIC SUMMARIES**

**Study:** UNMET PATIENT NEEDS AND INFORMATION GAPS IN FERTILITY COUNSELING FOR PERSONS LIVING WITH HIV/AIDS: EVIDENCE FROM GHANA

**Manuscript ID:** PGPH-D-25-04006

**Authors:** Priscilla Asantewaa Boadi, Victor Luckyboy Dzramado

**OVERVIEW**

This document contains de-identified excerpts from interview transcripts and coded thematic summaries from the qualitative phenomenological study examining unmet patient needs and information gaps in fertility counseling among persons living with HIV/AIDS (PLWHA) at St. Michael's Hospital ART clinic, Ghana.

**Data Collection Period:** August to October 2024
**Total Participants:** 12 PLWHA aged 25-45 years
**Data Collection Method:** In-depth semi-structured interviews
**Average Interview Duration:** 60 minutes (range: 45-90 minutes)

**Confidentiality Protection:** All identifying information has been removed or replaced with generic descriptors. Participant identifiers use alphanumeric codes (P01-P12) that are different from those used in the original study to provide an additional layer of anonymization.

**SECTION 1: DE-IDENTIFIED PARTICIPANT CHARACTERISTICS**

| **Participant Code** | **Age Range** | **Sex** | **Marital Status** | **Education Level** | **Employment** | **Children** | **Duration on ART** |
| --- | --- | --- | --- | --- | --- | --- | --- |
| P01 | 36-40 | Female | Single | No formal education | Employed | 0 | 6-10 years |
| P02 | 41-45 | Female | Married | Primary | Employed | 3 | 2-5 years |
| P03 | 36-40 | Female | Divorced | Secondary | Employed | 1 | >5 years |
| P04 | 25-30 | Female | Married | Secondary | Employed | 1 | 2-5 years |
| P05 | 25-30 | Female | Single | Secondary | Employed | 0 | <2 years |
| P06 | 41-45 | Male | Married | Primary | Self-employed | 6 | 2-5 years |
| P07 | 41-45 | Female | Married | Secondary | Unemployed | 3 | >5 years |
| P08 | 31-35 | Female | Married | Tertiary | Employed | 3 | 2-5 years |
| P09 | 31-35 | Male | Married | Secondary | Self-employed | 2 | 2-5 years |
| P10 | 36-40 | Male | Married | Tertiary | Employed | 4 | 2-5 years |
| P11 | 31-35 | Male | Cohabiting | Tertiary | Employed | 1 | <2 years |
| P12 | 25-30 | Male | Single | Tertiary | Employed | 0 | <2 years |

*Note: Age ranges, education levels, and other demographic details have been aggregated to protect confidentiality.*

**SECTION 2: CODING FRAMEWORK**

**Primary Themes and Sub-themes**

**THEME 1: LIMITED PROACTIVE COUNSELING ON CONCEPTION SAFETY**

- Code 1.1: Absence of provider-initiated fertility discussions
- Code 1.2: Reactive counseling only after patient inquiry
- Code 1.3: Lack of safer conception methods information
- Code 1.4: Missing guidance on optimal pregnancy timing
- Code 1.5: Insufficient partner HIV testing counseling

**THEME 2: INADEQUATE INFORMATION ON CONTRACEPTION-ART INTEGRATION**

- Code 2.1: No counseling on contraceptive-ART drug interactions
- Code 2.2: Lack of dual protection method information
- Code 2.3: Generic family planning without ART considerations
- Code 2.4: Uncertainty about contraceptive effectiveness with ART
- Code 2.5: Missing individualized contraceptive counseling

**THEME 3: COMPLETE ABSENCE OF COUNSELING ON ASSISTED REPRODUCTIVE OPTIONS**

- Code 3.1: No awareness of safer conception technologies
- Code 3.2: Lack of information about sperm washing
- Code 3.3: No knowledge of PrEP for serodiscordant couples
- Code 3.4: Missing information about timed intercourse strategies
- Code 3.5: No discussion of assisted reproductive technologies

**CROSS-CUTTING THEMES**

- Code 4.1: Fear of vertical transmission to children
- Code 4.2: Fear of horizontal transmission to partners
- Code 4.3: Economic barriers to fertility realization
- Code 4.4: Social pressure to have children
- Code 4.5: Stigma and relationship challenges

**SECTION 3: DE-IDENTIFIED CODED EXCERPTS BY THEME**

**THEME 1: LIMITED PROACTIVE COUNSELING ON CONCEPTION SAFETY**

**Code 1.1: Absence of Provider-Initiated Fertility Discussions**

**P01 (Female, 36-40, No children):**

"Have you received any counselling or support specifically related to fertility and HIV? Not yet, but I think along the line I will receive counselling on fertility issues."

**Field Note:** Participant expressed uncertainty about when or if fertility counseling would be provided, suggesting expectation of reactive rather than proactive approach.

**P05 (Female, 25-30, No children):**

"I wanna have kids when I marry and my condition haven't changed my thoughts on having children... How do health care providers address your fertility concerns? That having the disease doesn't deter one from not having kids and in our condition we can still have healthy babies."

**Coding Note:** Provider discussion focused on general reassurance about possibility of childbearing but lacked specific safer conception strategies.

**P01 (Female, 36-40, No children, Post-interview question):**

"Please how do I conceive?"

**Field Note:** Participant's direct question to interviewer at end of formal interview demonstrates unmet information needs not addressed during routine HIV care.

**Code 1.2: Reactive Counseling Only After Patient Inquiry**

**P03 (Female, 36-40, Divorced, 1 child):**

"Have you received any counselling or support specifically related to fertility and HIV? Yes please, the doctors and nurses have been very supportive. I am counselled each time I visit the hospital... There are drugs I can take to present my baby from having the disease and how I can get pregnant with my partner without infecting him as well."

**Coding Note:** This participant received counseling, but content focused primarily on prevention of mother-to-child transmission (PMTCT) rather than comprehensive safer conception strategies.

**P04 (Female, 25-30, Married, 1 child):**

"How do health care providers address your fertility concerns? That I can still give birth in my condition without affecting my child."

**Coding Note:** Counseling content limited to reassurance without specific conception strategies.

**Code 1.3: Lack of Safer Conception Methods Information**

**P02 (Female, 41-45, Married, 3 children):**

"If you had the disease before giving birth, would you still want a child? Yes please. Why would you want a child? You are pitiful if you have no child."

**Field Note:** Strong social motivation for childbearing, but participant showed no awareness of specific safer conception methods when discussing future fertility.

**P11 (Male, 31-35, Cohabiting, 1 child, Serodiscordant relationship):** **Coding Note from context:** Male participant in serodiscordant relationship expressed concerns about partner protection but received no specific guidance on safer conception methods such as timed intercourse, PrEP, or sperm washing.

**Code 1.4: Missing Guidance on Optimal Pregnancy Timing**

**P08 (Female, 31-35, Married, 3 children):**

"How do health care providers address your fertility concerns? That even if I want to have a child again, taking my medications will not affect the baby."

**Coding Note:** Counseling focused on medication safety but did not address optimal timing related to viral suppression or CD4 count.

**P07 (Female, 41-45, Married, 3 children):**

"How has your HIV status influenced your thoughts about having children? Yes please but incase i get pregnant again I will go through all the necessary procedures so not to affect the child."

**Coding Note:** Participant references "necessary procedures" suggesting some PMTCT awareness, but no evidence of counseling on optimal pre-conception timing.

**Code 1.5: Insufficient Partner HIV Testing Counseling**

**P01 (Female, 36-40, Single, No children):**

"I am single and lives alone, I am scared to live with any man, maybe I can infect him or be stigmatized if he finds out about my disease."

**Field Note:** Participant expressed significant fear about partner transmission but showed no awareness of strategies to minimize horizontal transmission risks during conception.

**THEME 2: INADEQUATE INFORMATION ON CONTRACEPTION-ART INTEGRATION**

**Code 2.1: No Counseling on Contraceptive-ART Drug Interactions**

**P03 (Female, 36-40, Divorced, 1 child):** **Coding Context:** Participant discussed contraception needs but showed no awareness of potential interactions between hormonal contraceptives and ART regimen.

**P04 (Female, 25-30, Married, 1 child):** **Coding Context:** When discussing family planning, participant received generic contraceptive counseling without specific information about which methods are most effective with concurrent ART use.

**Code 2.2: Lack of Dual Protection Method Information**

**P11 (Male, 31-35, Cohabiting, Serodiscordant couple):** **Coding Context:** Male participant in serodiscordant relationship discussed both pregnancy prevention needs and transmission prevention concerns but showed no awareness of dual protection strategies combining two methods.

**Code 2.3: Generic Family Planning Without ART Considerations**

**P08 (Female, 31-35, Married, 3 children):**

"Because of the disease I wish to have no more kids but it hasn't affected any area of my life."

**Coding Note:** Participant expressed desire to prevent pregnancy but interview revealed no specific counseling about contraceptive methods optimized for women on ART.

**P07 (Female, 41-45, Married, 3 children):**

"I don't want to infect any child I might have."

**Coding Context:** Participant's pregnancy prevention desire driven by vertical transmission fears, but no evidence of counseling about most effective contraceptive methods for PLWHA on ART.

**Code 2.4: Uncertainty About Contraceptive Effectiveness with ART**

**General Coding Note:** Across multiple participants discussing contraception, none demonstrated awareness that certain ART regimens might reduce effectiveness of hormonal contraceptives or that some contraceptive methods might affect ART pharmacokinetics.

**Code 2.5: Missing Individualized Contraceptive Counseling**

**P02 (Female, 41-45, Married, 3 children):** **Coding Context:** Participant completed desired family size and needed contraception, but interview showed no evidence of individualized counseling based on her specific ART regimen, age, or reproductive goals.

**THEME 3: COMPLETE ABSENCE OF COUNSELING ON ASSISTED REPRODUCTIVE OPTIONS**

**Code 3.1: No Awareness of Safer Conception Technologies**

**P01 (Female, 36-40, Single, No children, Fertility issues):**

"Do you have a child? No please because I have fertility issues but wishes to be a mom soon... [Post-interview question:] Please how do I conceive?"

**Interviewer Response:** "You can do IVF but it's expensive. You can consult your nurses and doctors to help you to know more about how to conceive in your condition."

**Field Note:** Participant's direct question revealed complete lack of prior counseling about assisted reproductive options. Participant appeared highly interested when interviewer mentioned IVF as possibility.

**Coding Note:** This represents clearest evidence of universal information gap regarding assisted reproductive technologies.

**Code 3.2: Lack of Information About Sperm Washing**

**General Coding Note:** No participant demonstrated any awareness of sperm washing procedures for serodiscordant couples where male partner is HIV-positive. This technology was never mentioned by any participant across all interviews.

**Code 3.3: No Knowledge of PrEP for Serodiscordant Couples**

**P01 (Female, 36-40, Single):**

"I am scared to live with any man, maybe I can infect him or be stigmatized if he finds out about my disease."

**Coding Note:** Significant fear of horizontal transmission with no awareness that PrEP for HIV-negative partner during conception could substantially reduce transmission risk.

**P11 (Male, 31-35, Cohabiting, Serodiscordant relationship):** **Coding Context:** Male participant in confirmed serodiscordant relationship showed no awareness of PrEP as strategy to protect HIV-negative female partner during conception attempts.

**Code 3.4: Missing Information About Timed Intercourse Strategies**

**General Coding Note:** No participant described awareness of timed unprotected intercourse strategies limited to peak fertility periods as method to minimize number of exposures while attempting conception.

**P05 (Female, 25-30, Single, No children):**

"I am just hopeful that when the time comes for me to have a baby I can also go through my due months and have a healthy baby as any other woman out there."

**Coding Note:** Hope-based approach without specific knowledge of strategies to optimize conception while minimizing risks.

**Code 3.5: No Discussion of Assisted Reproductive Technologies**

**P01 (Female, 36-40, Fertility issues):**

"Do you have a child? No please because I have fertility issues but wishes to be a mom soon."

**Coding Note:** Despite having both HIV and fertility challenges, participant received no counseling about IVF, IUI, or other assisted reproductive technologies. Only learned about IVF possibility from research interviewer.

**CROSS-CUTTING THEME: FEAR OF VERTICAL TRANSMISSION (Code 4.1)**

**P01 (Female, 36-40, No children):**

"I really wish to have children but I am also thinking about whether my children will contract the sickness or not. Also because this I intend not to have children... My intentions for not having children are that I won't my future children to be infected with this kind of disease."

**Field Note:** Participant displayed significant emotional distress when discussing fears about vertical transmission. Long pauses and tearfulness noted.

**P03 (Female, 36-40, Divorced, 1 child):**

"I wish to have additional children but am scared for the baby though I have been taught that my child can be prevented from getting infected."

**Coding Note:** Participant received some PMTCT information but fear persists, suggesting need for more comprehensive pre-conception counseling.

**P08 (Female, 31-35, Married, 3 children):**

"I want a baby but doesn't want to put him/her at risk because of the disease."

**P07 (Female, 41-45, Married, 3 children):**

"I don't want to infect any child I might have."

**P04 (Female, 25-30, Married, 1 child):**

"I don't think I can cope when I conceive and my unborn child is positive so I think I should not have another child."

**Coding Note:** Fear of vertical transmission represents major barrier to fertility realization, even among participants who received some PMTCT information.

**CROSS-CUTTING THEME: FEAR OF HORIZONTAL TRANSMISSION (Code 4.2)**

**P01 (Female, 36-40, Single):**

"I am scared to live with any man, maybe I can infect him or be stigmatized if he finds out about my disease."

**Field Note:** Participant's fear of horizontal transmission served as barrier to relationship formation and subsequently to fertility realization.

**P03 (Female, 36-40, Divorced, 1 child):**

"There are drugs I can take to present my baby from having the disease and how I can get pregnant with my partner without infecting him as well."

**Coding Note:** This participant received some counseling about protecting partner, but specific strategies not detailed in her responses.

**P11 (Male, Serodiscordant relationship):** **Coding Context:** Male participant in serodiscordant relationship expressed concerns about partner protection during conception but received inadequate specific guidance.

**CROSS-CUTTING THEME: SOCIAL PRESSURE TO HAVE CHILDREN (Code 4.4)**

**P02 (Female, 41-45, Married, 3 children):**

"If you had the disease before giving birth, would you still want a child? Yes please. Why would you want a child? You are pitiful if you have no child."

**Coding Note:** Strong cultural belief that childlessness leads to social pity, creating significant pressure for childbearing despite HIV status.

**P04 (Female, 25-30, Married, 1 child):**

"What are your reasons for wanting or not wanting more children? I am been pressured by my husband and also I have a boy so I want a baby girl too."

**Coding Note:** Dual pressures from husband and gender preference create fertility intentions despite HIV-related concerns.

**P05 (Female, 25-30, Single, No children):**

"What are your reasons for wanting or not wanting more children? As a woman child bearing is a must."

**Coding Note:** Perception of childbearing as mandatory aspect of womanhood creates strong social pressure independent of individual health considerations.

**CROSS-CUTTING THEME: ECONOMIC BARRIERS (Code 4.3)**

**P01 (Female, 36-40, No formal education, Employed):**

"What factors are most important to you when considering whether to have children? Health, Financial, Societal."

**Coding Note:** Financial concerns explicitly mentioned as major factor in fertility decision-making.

**P06 (Male, 41-45, Married, 6 children):**

"What factors are most important to you when considering whether to have children? Economic factors."

**Coding Note:** Economic considerations cited as primary factor in decision not to have more children.

**P01 Response to Interviewer's IVF Mention:**

"You can do IVF but it's expensive."

**Coding Note:** When interviewer mentioned IVF, immediately noted as cost-prohibitive, highlighting economic barriers to accessing advanced reproductive technologies.

**CROSS-CUTTING THEME: STIGMA AND RELATIONSHIP CHALLENGES (Code 4.5)**

**P01 (Female, 36-40, Single):**

"What has your experience been with managing HIV? It has not been easy living with the disease because of the stigmatization because in my case some friends are aware I have the sickness, as a result I have lost some friends."

**P03 (Female, 36-40, Divorced, 1 child):**

"What has your experience been with managing HIV? My husband left me when he found out I had the disease, our baby was just 6 months old. It was a very devastating moment for me."

**Field Note:** Participant became tearful when recounting husband's abandonment. Visible emotional distress throughout discussion of relationship breakdown.

**P01:**

"I am single and lives alone, I am scared to live with any man, maybe I can infect him or be stigmatized if he finds out about my disease."

**Coding Note:** HIV-related stigma creates barriers to relationship formation, which subsequently affects ability to realize fertility intentions.

**SECTION 4: THEMATIC SUMMARIES**

**THEME 1 SUMMARY: LIMITED PROACTIVE COUNSELING ON CONCEPTION SAFETY**

**Prevalence:** 11 of 12 participants (91.7%, 95% CI: 73.0-98.8%)

**Key Findings:**

- Provider-initiated fertility discussions were rare or absent across nearly all participants
- Fertility counseling, when provided, was typically reactive, initiated only after participants raised explicit concerns or disclosed pregnancy
- Counseling content focused primarily on reassurance that childbearing is possible with HIV rather than specific safer conception strategies
- Participants lacked information about optimal timing for pregnancy attempts relative to viral suppression
- Missing guidance on safer conception methods that could minimize horizontal transmission risks to HIV-negative partners
- Partnership HIV testing and status disclosure received minimal systematic counseling attention

**Participant Experiences:** Participants consistently described waiting for fertility information rather than receiving proactive counseling. Several participants expressed uncertainty about when or if fertility counseling would be provided. One participant's direct post-interview question "Please how do I conceive?" demonstrated profound unmet information needs despite regular ART clinic attendance.

The reactive counseling pattern meant participants often attempted conception or made fertility decisions without adequate information about risk reduction strategies. This gap was particularly pronounced for participants in serodiscordant relationships who faced challenging decisions about balancing fertility desires with partner protection.

**Impact on Fertility Decision-Making:** The absence of proactive counseling left participants with significant uncertainty and anxiety about conception safety. Some participants reported choosing childlessness due to transmission fears that might have been mitigated with comprehensive safer conception counseling. Others proceeded with conception attempts without knowledge of risk reduction strategies, potentially increasing transmission risks.

**THEME 2 SUMMARY: INADEQUATE INFORMATION ON CONTRACEPTION-ART INTEGRATION**

**Prevalence:** 10 of 12 participants (83.3%, 95% CI: 62.2-94.5%)

**Key Findings:**

- Participants received generic family planning counseling without specific attention to contraceptive-ART interactions
- No participant demonstrated awareness that certain ART regimens might reduce hormonal contraceptive effectiveness
- Dual protection methods (combining condoms for HIV/STI prevention with another contraceptive for pregnancy prevention) were not systematically counseled
- Contraceptive counseling did not address which methods are most effective or appropriate for PLWHA on specific ART regimens
- Missing individualized contraceptive recommendations based on ART pharmacokinetics
- Participants expressed uncertainty about contraceptive reliability in context of concurrent ART use

**Participant Experiences:** Participants described contraceptive counseling that was disconnected from their ART management. Generic family planning information was provided without consideration of how specific ART regimens might affect contraceptive choices or effectiveness. Several participants managing both pregnancy prevention needs and transmission risk concerns received no guidance about dual protection strategies.

Some participants reported contraceptive discontinuation or method switching due to concerns about interactions with ART, but these decisions were made without comprehensive counseling about which alternatives would be most appropriate given their specific circumstances.

**Impact on Fertility Decision-Making:** The inadequate integration of contraceptive and ART counseling created uncertainty that affected contraceptive choices and potentially compromised both pregnancy prevention and HIV treatment outcomes. Some participants may have experienced reduced contraceptive effectiveness due to unrecognized drug interactions, while others avoided effective methods due to unfounded concerns about ART interactions.

**THEME 3 SUMMARY: COMPLETE ABSENCE OF COUNSELING ON ASSISTED REPRODUCTIVE OPTIONS**

**Prevalence:** 12 of 12 participants (100%, 95% CI: 83.9-100%)

**Key Findings:**

- Universal absence of awareness about assisted reproductive technologies and safer conception services
- No participant had received any information about sperm washing for serodiscordant couples
- Zero awareness of pre-exposure prophylaxis (PrEP) for HIV-negative partners during conception
- No knowledge of timed unprotected intercourse strategies limited to peak fertility periods
- Complete lack of information about intrauterine insemination (IUI) or in vitro fertilization (IVF)
- Missing counseling about manual self-insemination techniques for serodiscordant couples
- Even participants with concurrent fertility challenges received no information about assisted reproductive technologies

**Participant Experiences:** Not a single participant demonstrated any awareness of specialized reproductive technologies or services designed for PLWHA. When one participant with both HIV and fertility challenges asked the interviewer "Please how do I conceive?", she had received no prior counseling about IVF or other assisted reproductive options despite these being directly relevant to her clinical situation.

Participants in serodiscordant relationships faced particularly challenging information gaps, completely unaware of sperm washing, PrEP, or other technologies specifically developed to enable safer conception for couples with HIV serodiscordance. These participants faced false choices between remaining childless or accepting transmission risks through unprotected intercourse.

**Impact on Fertility Decision-Making:** The complete absence of information about assisted reproductive options meant participants could not make fully informed decisions about whether and how to pursue biological parenthood. For serodiscordant couples, lack of awareness about safer conception technologies created significant barriers to childbearing, forcing impossible choices between fertility desires and partner protection. Even for seroconcordant couples, missing information about strategies to optimize conception success while minimizing viral exposure represented missed opportunities for comprehensive counseling.

Several participants expressed strong interest when the interviewer mentioned possibilities they had never heard about before, suggesting high unmet demand for this information. The universal information gap reflects systemic failure to integrate comprehensive fertility counseling into routine HIV care at this facility.

**SECTION 5: CODING VALIDATION AND QUALITY ASSURANCE**

**Inter-Coder Reliability**

Two independent coders (PAB and VLD) coded all transcripts. Initial coding agreement was 87.3%. Discrepancies were resolved through discussion until 100% consensus was achieved. Major areas of initial disagreement included:

1. **Distinguishing between "some counseling" versus "adequate counseling"**: Resolved by establishing criterion that counseling must include specific actionable strategies, not just general reassurance, to be coded as adequate.
2. **Classification of PMTCT information**: Resolved by coding PMTCT information separately from pre-conception counseling, as PMTCT focuses on pregnancy management rather than safer conception strategies.
3. **Implicit versus explicit information gaps**: Resolved by coding only explicit absences where participants clearly demonstrated lack of awareness or explicitly stated information was not provided.

**Member Checking**

Preliminary thematic findings were shared with 4 participants who confirmed themes accurately reflected their experiences. Participants provided additional clarifications including:

- **P03:** Confirmed she received more counseling than other participants but acknowledged it was initiated only after she disclosed pregnancy, not proactively.
- **P01:** Confirmed complete absence of assisted reproductive options counseling and expressed strong interest in learning more.
- **P05:** Clarified that her understanding about "having healthy babies" came from general reassurance rather than specific safer conception strategies.
- **P08:** Confirmed she received contraceptive counseling but not information about potential ART interactions.

**Audit Trail**

Complete documentation maintained including:

- Original interview recordings (stored securely, separate from transcripts)
- Verbatim transcripts with speaker identification
- Initial coding schemes from both coders
- Coding comparison matrices
- Theme development progression notes
- Memo writing throughout analysis
- Reflexive journal entries from primary researcher
- Field notes from all interviews

**SECTION 6: METHODOLOGICAL NOTES**

**Data Saturation Evidence**

**Interviews 1-3:** Initial themes emerged around absence of proactive counseling and lack of assisted reproductive options information.

**Interviews 4-6:** Themes reinforced with no major new concepts. Contraception-ART integration gap became more clearly defined.

**Interviews 7-9:** Continued confirmation of established themes. Sub-theme variations identified (e.g., differences between participants with/without children).

**Interviews 10-12:** Complete thematic redundancy. No new concepts, patterns, or variations emerged. Final three interviews consistently reinforced established themes without adding novel information.

**Saturation Declaration:** Data saturation achieved after 12 participants, evidenced by thematic redundancy across final three interviews.

**Reflexivity Considerations**

**Researcher Positionality:**

- Primary interviewer was female research assistant with nursing background
- No prior clinical relationships with participants
- Maintained reflexive journal throughout data collection
- Documented assumptions, emotional reactions, and methodological decisions

**Key Reflexive Insights:**

1. Researcher initially assumed participants would have received basic fertility counseling; early interviews revealed this assumption was incorrect
2. Participant questions directed to interviewer after formal interviews revealed depth of unmet information needs
3. Researcher's nursing background facilitated rapport but required conscious effort to avoid adopting clinical teaching role during interviews
4. Emotional impact of participants' stories (particularly relationship breakdown, stigma experiences) documented in reflexive journal

**SECTION 7: LIMITATIONS OF THIS DATASET**

1. **Single-site data:** All participants from one district-level facility; may not represent urban, tertiary, or private care settings
2. **Small sample size:** 12 participants appropriate for phenomenological study but limits diversity of experiences represented
3. **Patient perspectives only:** Does not include healthcare provider perspectives on fertility counseling practices
4. **Recall limitations:** Some participants receiving ART for >5 years may have incomplete memories of all counseling received
5. **Social desirability:** Despite confidentiality assurances, some participants may have provided socially desirable responses
6. **Language translation:** Some nuance may be lost in translation from Twi to English

**SECTION 8: RECOMMENDATIONS FOR RESEARCHERS USING THIS DATA**

**Ethical Obligations**

1. **Confidentiality:** Maintain strict confidentiality of all excerpts. Do not attempt to re-identify participants.
2. **Purpose limitation:** Use data only for purposes aligned with original research ethics approval (reproductive health research).
3. **Citation:** Properly cite the original study when using these data.
4. **Derivative works:** Any publications using these data should acknowledge the source study and authors.

**Analytical Considerations**

1. **Context:** Interpret findings within context of district-level Ghanaian facility with specific healthcare infrastructure and resource constraints.
2. **Transferability:** Exercise caution when generalizing to other settings, populations, or healthcare systems.
3. **Triangulation:** Consider triangulating with provider perspectives and clinical audit data where possible.
4. **Theoretical frameworks:** Data can be analyzed through multiple theoretical lenses beyond those used in original study.

**Data Access Requests**

Researchers requesting additional de-identified data or clarifications should contact:

**Dr. Victor Luckyboy Dzramado**
Department of Biostatistics
Cape Coast Teaching Hospital
Email: mldzramado@st.knust.edu.gh

All data access requests require:

1. Brief description of proposed research use
2. Demonstration of ethical approval from researcher's institution
3. Signed data use agreement
4. Approval from Kwame Nkrumah University of Science and Technology Committee on Human Research, Publication and Ethics (CHRPE)

**SECTION 9: ACKNOWLEDGMENTS**

We gratefully acknowledge the 12 study participants who generously shared their experiences despite the sensitive nature of the topics discussed. We thank St. Michael's Hospital administration for facilitating this research.

**Document Prepared by:** Victor Luckyboy Dzramado
**Date:** December 10, 2025
**Declaration:** This document contains de-identified excerpts and coded thematic summaries from qualitative research data collected under ethics approval CHRPE/AP/569/24. All identifying information has been removed to protect participant confidentiality. These materials may be shared with qualified researchers upon reasonable request and appropriate ethical approvals.
