# Supplementary material for: Unmet patient needs and information gaps in fertility counseling for persons living with HIV/AIDS: Evidence from Ghana
Source: PLOS Glob Public Health. 2026 Apr 16;6(4):e0006017. doi: 10.1371/journal.pgph.0006017 (PMC13086305; doi:10.1371/journal.pgph.0006017)
Supplement: Attachment — Submitted filename: Response to Reviewers.docx [file pgph.0006017.s003.docx]

**RESPONSE TO REVIEWERS**

**Manuscript ID:** PGPH-D-25-04006

**Title:** UNMET PATIENT NEEDS AND INFORMATION GAPS IN FERTILITY COUNSELING FOR PERSONS LIVING WITH HIV/AIDS: EVIDENCE FROM GHANA

**Authors:** Priscilla Asantewaa Boadi, Victor Luckyboy Dzramado

Dear Dr. Hossain and Reviewers,

We thank you for the constructive feedback on our manuscript. We have carefully addressed all comments raised by the academic editor and both reviewers. Below is a detailed point-by-point response showing how we have revised the manuscript to address each concern.

**RESPONSE TO JOURNAL REQUIREMENTS**

| **Requirement** | **Original Issue** | **Our Response** | **Location in Revised Manuscript** |
| --- | --- | --- | --- |
| **1. Competing Interests Statement** | Complete competing interests statement needed, including any COIs declared by co-authors | We have added a complete Competing Interests statement. Both authors declare no competing interests exist. | **Page 28, Declarations section:** "The authors declare that they have no competing interests." |
| **2. Financial Disclosure Statement** | Detailed financial disclosure statement needed in full sentences with exact wording for publication | We have added a complete Financial Disclosure statement in full sentences. | **Page 28, Declarations section:** "The authors received no specific funding for this work." |
| **3. Data Availability Statement** | Statement does not fully meet PLOS requirements. Need to: (a) better justify restriction, (b) specify what de-identified materials can be shared, (c) clearly outline access conditions, (d) identify data custodian(s), (e) provide clear contact address | We have completely revised the Data Availability statement to address all five requirements: (a) Detailed ethical justification for restrictions citing participant confidentiality, lack of consent for public sharing, and small catchment area risks; (b) Specified that de-identified excerpts and coded thematic summaries are available; (c) Outlined clear access conditions requiring ethics approval and reasonable request evaluation; (d) Identified corresponding author (VLD) as data custodian; (e) Provided email contact (mldzramado@st.knust.edu.gh) for data access requests | **Page 28, Declarations section, Data Availability:** Complete revised statement spanning 8 lines with all required elements |
| **4. Supporting Information Files** | Supporting Information legends listed but no files uploaded | We have prepared Supporting Information files for upload: **S1:** COREQ Checklist (32-item completed checklist); **S2:** Demographic table (included in main manuscript as Table 1) | Supporting files prepared for upload with submission |

**RESPONSE TO REVIEWER #1 COMMENTS**

| **Comment** | **Reviewer Concern** | **Our Response** | **Location in Revised Manuscript** |
| --- | --- | --- | --- |
| **1. Methodological and Analytical Transparency** |  |  |  |
| 1a. Rationale for sample size and saturation | Further clarification needed regarding rationale for sample size and saturation | We have added a comprehensive new subsection (approximately 400 words) titled "Sample Size and Saturation" that: (1) Explains qualitative phenomenological sample size determination principles; (2) Cites appropriate literature on data saturation (Guest et al., Fusch & Ness, Saunders et al.); (3) Describes our iterative approach to saturation assessment; (4) Details how saturation was achieved after 12 participants with thematic redundancy across final 3 interviews; (5) Justifies final sample size alignment with phenomenological research recommendations | **Pages 8-9, Methods section, Sample Size and Saturation subsection:** Complete new subsection with 5 paragraphs and 11 references (references 37-46, Francis et al., Hennink et al., Malterud et al., Mason, Morse, Vasileiou et al.) |
| 1b. Coding procedures and reflexivity | Further clarification needed regarding coding procedures and reflexivity | We have added two major enhancements: **(1) Coding Process subsection** (approximately 350 words) describing: open coding procedures with line-by-line analysis; axial coding with constant comparison; selective coding integrating categories into themes; use of NVivo software version 12. **(2) Reflexivity subsection** (approximately 250 words) detailing: researcher credentials and experience; absence of prior relationships with participants; reflexive journaling throughout research; critical examination of researcher positioning and assumptions | **Pages 11-12, Data Analysis section:** New "Coding Process" subsection with detailed multi-stage coding description and 10 references (references 55-65); **Page 7, Methods section:** New "Reflexivity and Researcher Positionality" subsection integrated into Sample Population section |
| **2. Reporting Guideline Compliance** |  |  |  |
| 2a. COREQ adherence | Manuscript does not explicitly state adherence to COREQ qualitative reporting guideline. Explicit reporting guideline alignment is a PLOS requirement | We have added explicit statement of COREQ adherence in multiple locations: (1) New opening paragraph in Methods section stating the study "adheres to the Consolidated Criteria for Reporting Qualitative Research (COREQ) guidelines" with full citation (Tong et al., 2007, reference 34); (2) Description of COREQ as "a 32-item checklist specifically developed for reporting interviews and focus groups"; (3) Reference to completed COREQ checklist in Supporting Information; (4) COREQ citation added to abstract methods | **Page 6, Methods section, Study Design and Reporting Guidelines:** Opening paragraph explicitly states COREQ adherence; **Page 2, Abstract, Methods:** "following COREQ guidelines" added; **Page 28, Supporting Information:** S1 COREQ Checklist referenced |
| **3. Ethics and Data Availability** |  |  |  |
| 3a. Ethical approval statement | Inclusion of ethical approval in abstract is appropriate; however, statement could be strengthened by clearly indicating informed consent was obtained, or briefly clarifying nature of approval. Should be more prominently and systematically presented | We have strengthened ethical statements throughout: **(1) Abstract:** Added "all participants provided written informed consent"; **(2) Methods - Ethical Considerations:** Expanded section to 3 full subsections (Informed Consent, Confidentiality and Privacy, Protection from Harm) totaling approximately 400 words with detailed description of consent procedures, voluntary participation, right to withdraw, language accessibility, and protection measures; **(3) Ethics Approval declaration:** Added comprehensive statement in Declarations including both ethics committees and Declaration of Helsinki compliance | **Page 2, Abstract:** "all participants provided written informed consent" added after ethics approval; **Pages 13-14, Methods:** Complete "Ethical Considerations" section with 3 detailed subsections; **Page 28, Declarations:** "Ethics Approval and Consent to Participate" with comprehensive statement including Declaration of Helsinki |
| 3b. Data availability statement | While statement is appropriate given sensitive data, does not fully meet PLOS requirements. Authors should: better justify restriction, specify what de-identified materials can be shared, clearly outline access conditions, identify data custodian(s), provide clear contact address | Comprehensively revised Data Availability statement (8 lines) now includes: **(1) Justification:** Detailed explanation of ethical restrictions, lack of public sharing consent, and small catchment area confidentiality risks; **(2) Shareable materials:** Specified "de-identified excerpts and coded thematic summaries" available; **(3) Access conditions:** Requires reasonable request, ethics approval from CHRPE, evaluation based on ethical appropriateness and scientific merit; **(4) Data custodian:** Corresponding author (VLD) clearly identified as data custodian; **(5) Contact:** Email mldzramado@st.knust.edu.gh provided for data access requests | **Page 28, Declarations section, Data Availability:** Complete 8-line statement with all five required elements explicitly addressed |
| **4. Discussion and Interpretation** |  |  |  |
| 4a. Deeper engagement with limitations | Discussion would benefit from deeper engagement with limitations including single-site study, small sample, and transferability | We have substantially expanded the Limitations subsection (now approximately 600 words with 6 distinct limitation paragraphs): **(1) Single-site limitation:** Acknowledged district-level facility may not represent urban, tertiary, or private settings; explicitly discussed transferability considerations; **(2) Small sample size:** Acknowledged 12 participants, while appropriate for phenomenology and achieving saturation, limits diversity of experiences; called for larger multi-site studies; **(3) Provider perspectives:** Acknowledged study only captured patient perspectives, noted need for complementary provider research; **(4) Cross-sectional design:** Noted limitation of single time-point data, called for longitudinal research; **(5) Social desirability bias:** Acknowledged potential for response bias despite confidentiality measures; **(6) Recall bias:** Noted memory limitations for long-term ART users | **Pages 24-25, Discussion section, Study Limitations:** Expanded subsection with 6 comprehensive limitation paragraphs, each with detailed discussion of implications and recommendations for future research |
| 4b. More cautious framing of implications | Some conclusions verge on generalization beyond study context. Need more cautious framing of implications for policy and clinical practice | We have systematically reframed all conclusions and implications with cautious, context-appropriate language: **(1) Abstract conclusion:** Changed from definitive recommendations to "This study reveals substantial unmet patient needs" at "this district-level facility" and states interventions "warrant further investigation"; **(2) Implications section:** Reframed as "potential areas for intervention development" requiring "systematic evaluation in this setting" with repeated use of "may warrant investigation," "might be worth exploring," "could potentially"; **(3) Main conclusion:** Extensively revised to emphasize findings are from "single-site study with 12 participants," states "conclusions about broader implications...should be drawn cautiously," and explicitly calls for "further research involving multiple sites, diverse settings, and larger sample sizes" before generalizing | **Page 2, Abstract:** Cautious language throughout conclusion; **Pages 23-24, Discussion, Implications section:** Every recommendation qualified with cautious framing; **Page 25, Discussion, Conclusion:** Completely rewritten with extensive caveats about generalizability and explicit calls for further research |
| **5. Language and Presentation** |  |  |  |
| 5a. Tightening language | Manuscript written in good academic English but would benefit from tightening, particularly in abstract and discussion | We have systematically tightened language throughout: **(1) Abstract:** Reduced redundancy, eliminated unnecessary qualifiers, streamlined sentence structure while maintaining all essential information; **(2) Discussion:** Condensed verbose passages, eliminated repetitive phrasing, improved transition sentences, maintained scholarly tone with increased precision; **(3) Throughout manuscript:** Removed unnecessary adverbs, consolidated related ideas, eliminated redundant explanations. No em-dashes used anywhere in manuscript per author requirements | **Throughout manuscript:** Systematic language tightening while preserving academic rigor and completeness. **Particularly:** Pages 1-2 (Abstract), Pages 20-26 (Discussion section) |

**RESPONSE TO REVIEWER #2 COMMENT**

| **Comment** | **Reviewer Concern** | **Our Response** | **Location in Revised Manuscript** |
| --- | --- | --- | --- |
| **Field notes analysis and contribution** | Authors state they collected field notes documenting nonverbal cues, emotional reactions, and contextual observations; however, unclear how data from these methods were analyzed or whether they contributed to results. Readers would benefit from greater clarity on how these data were analyzed and what insights they generated, particularly to support replication in similar contexts | We have added comprehensive clarification of field notes' role in analysis and contribution to findings: **(1) Data Collection section:** Added new paragraph (approximately 150 words) under "Field Notes and Contextual Observations" subsection explaining: field notes documented specific types of observations (facial expressions, body language, emotional responses, environmental conditions); field notes were reviewed immediately after each interview; specific examples of field notes applications; **(2) Data Analysis section:** Added explicit statement that "Field notes were reviewed immediately following each interview and integrated into the analytical process during transcript coding"; **(3) Results section:** Added specific field note observations integrated into each sub-theme narrative (e.g., "Field notes documented that this participant displayed visible emotional distress when recounting her experience, with long pauses and tearfulness" for Sub-theme 1; similar integrations in Sub-themes 2 and 3); **(4) Methods:** Clarified field notes "provided important contextual information that enriched data interpretation, capturing dimensions of participants' experiences that might not be fully evident in verbal transcripts alone" with specific examples of what was captured | **Page 10, Methods, Data Collection:** New paragraph in "Field Notes and Contextual Observations" subsection with detailed explanation of field notes methodology and analytical integration (approximately 250 words total in this subsection with 4 new references: 50-53); **Page 12, Methods, Data Analysis:** Explicit statement about field notes integration into coding; **Pages 15-20, Results:** Multiple specific field note observations integrated into each sub-theme narrative showing how field notes enriched interpretation (approximately 6-8 field note observations distributed across the three sub-themes) |

**SUMMARY OF MAJOR REVISIONS**

We have made the following major revisions to strengthen the manuscript:

**1. Enhanced Methodological Transparency (≈1,200 new words)**

- Added comprehensive Sample Size and Saturation subsection
- Added detailed Coding Process description with multi-stage approach
- Added Reflexivity and Researcher Positionality subsection
- Clarified field notes collection, analysis, and contribution to findings

**2. Explicit COREQ Compliance**

- Added COREQ adherence statement in Methods
- Referenced COREQ in Abstract
- Prepared completed COREQ checklist as Supporting Information S1

**3. Strengthened Ethics and Data Availability (≈500 new words)**

- Expanded Ethical Considerations section to 3 detailed subsections
- Added informed consent clarification throughout
- Completely revised Data Availability statement with all PLOS-required elements

**4. Improved Discussion Rigor (≈800 new words)**

- Substantially expanded Limitations section with 6 comprehensive paragraphs
- Systematically reframed all implications with cautious, context-appropriate language
- Extensively revised conclusion to emphasize study limitations and need for further research

**5. Added Required Declarations**

- Competing Interests statement
- Financial Disclosure statement
- Complete Authors' Contributions statement

**6. Language Refinement**

- Tightened abstract and discussion
- Eliminated redundancy throughout
- No em-dashes used (per author requirements)

**7. Enhanced Results Integration**

- Integrated field note observations throughout Results section
- Improved flow and connections between themes
- Maintained all three tables with proper referencing

We believe these revisions have substantially strengthened the manuscript's methodological rigor, reporting transparency, and appropriate contextualization of findings. The manuscript now fully meets PLOS Global Public Health's publication criteria and reporting guidelines.

We thank the reviewers for their constructive feedback, which has significantly improved the quality and rigor of our work.

Sincerely,

……………………….

Victor Luckyboy Dzramado

Corresponding Author

Research Assistant

University of Cape Coast

Cape Coast, Ghana
