# Supplementary material for: Unmet patient needs and information gaps in fertility counseling for persons living with HIV/AIDS: Evidence from Ghana
Source: PLOS Glob Public Health. 2026 Apr 16;6(4):e0006017. doi: 10.1371/journal.pgph.0006017 (PMC13086305; doi:10.1371/journal.pgph.0006017)
Supplement: Attachment — Submitted filename: UNMET PATIENT NEEDS_peer review_Eduarda Gusmao.docx [file pgph.0006017.s004.docx]

1. **Summary of the research** In your own words, summarize the main research question, claims, and conclusions of the study. Provide context for how this research fits within the existing literature. Discuss the manuscript’s strengths and weaknesses and your overall recommendation.

Very interesting study, bringing a different perspective into reproductive rights and choices unmet needs. Most research focuses on existing medication/stock outs, services integration, client’s methods preferences as main factors impacting access to reproductive care. However, even with medication is available, services are integrated and preferred methods are available, other gaps may impact access to timely and proper sexual and reproductive counseling, and understanding clients perspective can inform policy development and adaptations to further reduce the reproductive health gaps among PLHIV.

The authors present a qualitative study aiming to identify information gaps and unmet needs from reproductive counseling as part of clinical follow-up at HIV clinic for people living with HIV (PLWH) in a semi-urban/rural district in Ghana. This study used a phenomenological approach, allowing understanding of clients lived experience to respond to the study aim and objectives. While using a semi-structured interview guide, the examples provided of questions asked show that the authors were careful to avoid leading and loaded questions, therefore preventing biased questions nudging the participants towards pre-concepted assumptions.

Major strengths:

- Relevant topic and interesting approach to understanding client’s views of gaps in one important area related to PLHIV and women’s health.
- Well structured paper, presenting properly the relevant findings and existing gaps in the literature associated with the theme, well positioning the paper.
- Detailed description of the study design and methodology, including detailed steps on data recording, collection, transcription and analysis, allowing for clear understanding and replication if necessary/of interest.
- Brings relevant information, bringing behavior aspects in addition to existing knowledge and structural aspects impacting access to reproductive health for PLHIV. Providing inputs to inform policy and guidelines development and program adaptation to address unmet needs of reproductive health for PLHIV.
- The paper acknowledges some important limitation of the study, including the limited scope and potentially non transferability of these findings to other settings, as well as potential recall bias.

Weakness:

- Not clear what are the national guidelines for reproductive counseling in Ghana, it would be interesting to cite the key elements and align with the aspects assessed.
- While the paper includes PrEP as one of the assisted reproductive options to ensure or reduce risk of horizontal infection of sexual partner, it misses presenting the indetectable equals intransmissable (I=I) concept as an alternative for safe conception.
- There is a wide focus on horizontal transmission to sexual partners as part of the reproductive rights, while limited information, in the background and discussion and conclusion, of the impact of reproductive rights/health unmet needs have on women’s health, maternal and neonatal mortality and on vertical transmission of HIV, some of the major topics related to maternal and child health in the region.

My recommendation is to proceed with paper submission after the issues below are considered and addressed.

2. **Examples and evidence Major issues**

Issues for consideration:

- The sub-themes “Limited Proactive Counseling on Conception Safety”. “Inadequate Information on Contraception-ART Integration” and “Complete Absence of Counseling on Assisted Reproductive” are clearly described in the paper methods and results section as themes identified as a result of the in-depth interviews and the grouping of themes brought by participants. However, in the paper abstract, background and objective, those three large areas are already presented as important gaps for proper reproductive counseling. This leads to the misconception that the study was biased to lead participants to agree with these pre-existing assumptions. Suggest reviewing the language in the paper introduction to reduce this perception and only present the sub-themes when presenting the results and conclusions.
- It would be interesting to understand if the findings regarding unmet needs and gap in information were similar (or not) when comparing responses from participants in stable relationships and the others (single and/or separated/divorced). As most of the study participants were in stable relationships, their responses may have skewed the findings for the overall population covered in the study, as clients not in stable relationships may have different perceptions.
- Aligned with one of the points described above under “weakness”, it would be important to provide information regarding reproductive health guidelines in Ghana as part of the paper, while still considering word count. It is not clear, for example, if the lack of counseling related to assisted reproductive options is associated with lack of knowledge by healthcare providers, as those options are not included in most national guidelines in the region.
- Suggest adding a note on why I=I was not included in the assessment for sub-theme 3 (assisted reproductive options), while PrEP was. The study was held in 2024 when I=I was widely disseminated.
- Suggest adding to the background, as well as the discussion and conclusion section, the importance and impact proper reproductive health access to women living with HIV would have on maternal mortality and vertical transmission of HIV, considering the importance those topics have in the region.
- Under “implication for practice and policy” section, brings interesting suggestions, such as training package, clients support tools, counseling training for providers, etc. However, as the paper lacks information on existing policies and materials, it is not clear if those are relevant. Understanding provider’s knowledge would be an interesting intervention as a follow-up, to understand if the gap is on behavior, knowledge or guidelines and material content. This is briefly cited under “limitation” section, however, not explored under other sections.

3. I have no additional comments and am available to review an updated version.
