# Supplementary material for: Unmet patient needs and information gaps in fertility counseling for persons living with HIV/AIDS: Evidence from Ghana
Source: PLOS Glob Public Health. 2026 Apr 16;6(4):e0006017. doi: 10.1371/journal.pgph.0006017 (PMC13086305; doi:10.1371/journal.pgph.0006017)
Supplement: Attachment — Submitted filename: Response to Reviewers 2.docx [file pgph.0006017.s005.docx]

**RESPONSE TO REVIEWERS**

**Manuscript ID:** PGPH-D-25-04006R2

**Title:** UNMET PATIENT NEEDS AND INFORMATION GAPS IN FERTILITY COUNSELING FOR PERSONS LIVING WITH HIV/AIDS: EVIDENCE FROM GHANA

**Authors:** Priscilla Asantewaa Boadi, Victor Luckyboy Dzramado

Dear Editor and Reviewer,

We sincerely thank you for the thorough and insightful review of our manuscript. Your constructive feedback has substantially strengthened our work. We have carefully addressed all comments and concerns raised. Below is a detailed point-by-point response showing how we have revised the manuscript.

**RESPONSE TO REVIEWER COMMENTS**

| **Comment Category** | **Reviewer Comment** | **Our Response** | **Location in Revised Manuscript** |
| --- | --- | --- | --- |
| **SUMMARY OF RESEARCH - STRENGTHS** | Reviewer acknowledged: Relevant topic and interesting approach; Well-structured paper with proper positioning; Detailed methodology allowing replication; Brings behavioral aspects to inform policy; Acknowledges important limitations | We thank the reviewer for recognizing these strengths. We have worked to maintain these qualities while addressing the identified weaknesses. | Throughout manuscript |
| **WEAKNESS #1: National Guidelines** | "Not clear what are the national guidelines for reproductive counseling in Ghana, it would be interesting to cite the key elements and align with the aspects assessed." | We have added a comprehensive new subsection titled **"Reproductive Health Guidelines and Context in Ghana"** that: (1) Describes Ghana's National HIV and AIDS Strategic Plan 2021-2025 and its emphasis on SRH-HIV integration; (2) Details Ghana Health Service Family Planning Protocols and Standards requirements; (3) Presents implementation gaps from Ghana's 2022 DHS showing only 28% of HIV-positive women receive comprehensive counseling; (4) Cites provider knowledge assessments showing <40% possess comprehensive knowledge; (5) Aligns study findings with these policy-practice gaps. Added 8 new references (32-39, 47-50) documenting Ghana's guidelines and implementation challenges. | **Pages 4-5, Introduction section, new subsection:** "Reproductive Health Guidelines and Context in Ghana" (approximately 400 words with 8 new citations) |
| **WEAKNESS #2: U=U/I=I Concept** | "While the paper includes PrEP as one of the assisted reproductive options, it misses presenting the indetectable equals intransmissable (I=I) concept as an alternative for safe conception." | We have comprehensively integrated U=U (Undetectable=Untransmittable) throughout the manuscript: **(1) Introduction:** Added paragraph explaining U=U concept, its endorsement since 2016, incorporation into Ghana guidelines in 2019, and limited awareness (<30% of PLWHA in Ghana). **(2) Methods:** No changes needed as we explored patient experiences without predetermined topics. **(3) Results - Table 2:** Added new row showing 0% awareness of U=U concept among all participants. **(4) Results - Theme 1:** Added new subsection "Missing Information on U=U for Safer Conception" with participant quotes demonstrating lack of awareness despite sustained viral suppression. **(5) Results - Table 4:** Repositioned U=U as first row to emphasize its importance as primary safer conception strategy. **(6) Discussion:** Added analysis of why U=U absence is particularly concerning given 2019 guideline incorporation and provider knowledge gaps. Added 12 new references on U=U (38-43, 51-53, 68-71). | **Introduction:** Pages 5, new paragraph on U=U in Ghana context; **Results - Table 2:** Page 16, new row added; **Results - Theme 1:** Pages 17-18, new subsection (approximately 300 words); **Results - Table 4:** Page 20, U=U as first row; **Discussion:** Pages 23-24, U=U implementation gaps discussed |
| **WEAKNESS #3: Maternal/Child Health Impact** | "There is a wide focus on horizontal transmission to sexual partners as part of the reproductive rights, while limited information, in the background and discussion and conclusion, of the impact of reproductive rights/health unmet needs have on women's health, maternal and neonatal mortality and on vertical transmission of HIV" | We have substantially enhanced maternal and child health content: **(1) Introduction:** Revised paragraph 5 to add: maternal mortality risk (8-fold higher for HIV-positive pregnant women), vertical transmission statistics (up to 40% occurs during pregnancy/delivery without appropriate prophylaxis), and how inadequate counseling contributes to both. **(2) Discussion:** Added new major subsection **"Implications for Maternal and Child Health Outcomes"** (approximately 600 words) covering: Ghana's maternal mortality ratio and HIV-positive women's elevated risk; how unintended pregnancies and inadequate pre-conception counseling increase maternal death risk; vertical transmission rates in Ghana (5-15% vs WHO target <5%); how proactive pre-conception counseling could reduce MTCT; U=U counseling implications for both horizontal and vertical transmission prevention; comprehensive pre-conception counseling as critical intervention. **(3) Conclusion:** Reframed to explicitly state counseling gaps affect "not only reproductive autonomy but also maternal mortality reduction and vertical HIV transmission prevention, critical public health priorities in Ghana and sub-Saharan Africa." Added 15 new references on maternal mortality, vertical transmission, and PMTCT (26-29, 57-73). | **Introduction:** Page 3, enhanced paragraph 5; **Discussion:** Pages 24-26, new subsection "Implications for Maternal and Child Health Outcomes" (approximately 600 words with 15 new citations); **Conclusion:** Page 28, explicit maternal-child health framing |
| **MAJOR ISSUE #1: Pre-determined Assumptions** | "The sub-themes are clearly described as themes identified from interviews. However, in the abstract, background and objective, those three large areas are already presented as important gaps for proper reproductive counseling. This leads to the misconception that the study was biased to lead participants to agree with pre-existing assumptions. Suggest reviewing language in the paper introduction to reduce this perception and only present sub-themes when presenting results and conclusions." | We have comprehensively revised the Abstract, Introduction, and Results to eliminate language suggesting predetermined assumptions: **(1) Abstract - Background:** Removed specific mention of three gap areas, now states general need for "understanding patient experiences and perspectives." **(2) Abstract - Objective:** Changed from "examining deficiencies in X, Y, Z" to "explored patient experiences and perceived needs...examining the content and quality of reproductive health information provision." **(3) Abstract - Results:** Now presents themes as findings that "emerged from participant experiences" rather than confirming predetermined gaps. **(4) Introduction:** Completely rewritten final two paragraphs to state: "Rather than testing predetermined assumptions about counseling gaps, this qualitative phenomenological approach allowed participants to describe their lived experiences and identify areas where they perceived insufficient information." Emphasizes the research "explored patient experiences" without specifying what gaps would be found. **(5) Results:** Added new introductory paragraph stating: "These themes emerged inductively from participants' accounts of their experiences rather than from predetermined assumptions about counseling deficiencies." **(6) Theme Titles:** Changed from "Limited Proactive Counseling" (assertive) to "Patient Experiences of Limited Provider-Initiated Conception Safety Discussions" (descriptive of what participants reported). | **Abstract:** Page 1, Background and Objective sections completely reframed; **Introduction:** Page 6, final two paragraphs rewritten to emphasize exploratory approach; **Results:** Page 15, new introductory paragraph added; **Theme Titles:** Pages 15, 18, 20, all three themes reframed as patient experiences rather than predetermined gaps |
| **MAJOR ISSUE #2: Relationship Status Comparison** | "It would be interesting to understand if findings regarding unmet needs and gap in information were similar (or not) when comparing responses from participants in stable relationships and the others (single/separated/divorced). As most study participants were in stable relationships, their responses may have skewed findings for overall population." | We have added comprehensive relationship status analysis: **(1) Methods:** Added new subsection **"Analysis by Relationship Status"** describing our approach to stratifying coded data by relationship status (married/cohabiting vs. single/divorced) and examining for patterns or variations. **(2) Results - Participant Characteristics:** Added new paragraph **"Comparison by Relationship Status"** presenting findings: both partnered (n=8) and unpartnered (n=4) participants showed consistent patterns of counseling gaps across all three themes. Quantitative comparison: proactive counseling gaps (87.5% vs. 100%), contraception-ART integration gaps (75.0% vs. 100%), assisted reproductive options gaps (100% vs. 100%). Single/divorced participants expressed additional stigma concerns, but fundamental information gaps were consistent. Concludes: "This consistency suggests counseling deficiencies represent systemic service delivery gaps rather than differential treatment based on partnership status." | **Methods:** Page 13, new subsection "Analysis by Relationship Status" (approximately 150 words); **Results:** Page 15, new paragraph in Participant Characteristics section (approximately 200 words) presenting comparative analysis findings |
| **MAJOR ISSUE #3: Provider Knowledge Gap** | "Under 'implication for practice and policy' section, brings interesting suggestions such as training package, clients support tools, counseling training for providers, etc. However, as the paper lacks information on existing policies and materials, it is not clear if those are relevant. Understanding provider's knowledge would be an interesting intervention as a follow-up, to understand if the gap is on behavior, knowledge or guidelines and material content. This is briefly cited under 'limitation' section, however, not explored under other sections." | We have substantially revised the Implications section and added provider knowledge context throughout: **(1) Introduction:** Ghana guidelines subsection now includes provider knowledge assessment data showing <40% of providers possess comprehensive knowledge, <15% aware of ART availability, only 23% routinely counsel about U=U despite 2019 guideline incorporation. Cites provider-reported barriers (lack of training materials, inadequate time, no standardized protocols). **(2) Discussion:** Added new subsection **"Findings in Context of Ghana's Reproductive Health Guidelines and Provider Knowledge"** (approximately 500 words) analyzing: policy-practice gaps, provider knowledge deficits, time allocation constraints, systemic factors beyond individual provider knowledge, need for multi-level interventions. **(3) Implications Section:** Completely rewritten to emphasize need for provider assessment BEFORE intervention design. New structure: "Provider Knowledge and Training Needs Assessment" (first priority), then conditional interventions ("Contingent on provider needs assessment findings..."), explicit acknowledgment that "our study documented patient-perceived gaps, but underlying causes remain unclear," ends with **"Implementation Research Priorities"** calling for provider studies. **(4) Limitations:** Enhanced seventh limitation emphasizing patient vs. provider perspectives and need for complementary research. | **Introduction:** Pages 4-5, provider knowledge data integrated into Ghana guidelines subsection; **Discussion:** Pages 22-23, new subsection "Findings in Context of Ghana's Guidelines and Provider Knowledge" (approximately 500 words); **Discussion - Implications:** Pages 26-27, completely rewritten section (approximately 700 words) prioritizing provider needs assessment; **Limitations:** Page 28, enhanced provider perspective limitation |
| **MAJOR ISSUE #4: Why U=U Not Included in Original Assessment** | "Suggest adding a note on why I=I was not included in the assessment for sub-theme 3 (assisted reproductive options), while PrEP was. The study was held in 2024 when I=I was widely disseminated." | We have clarified this throughout: **(1) Methods clarification:** Our phenomenological approach explored participants' lived experiences without predetermined topics. We asked open-ended questions about safer conception information received, and participants described what they knew/didn't know. Neither U=U nor PrEP were specifically probed - both emerged (or failed to emerge) from participants' own accounts. **(2) Results presentation:** Now shows U=U FIRST in Table 4 as the primary safer conception strategy that should have been known. The universal absence (0% awareness) demonstrates this was the most critical gap - U=U should be foundational knowledge for all virally suppressed PLWHA in 2024. **(3) Discussion emphasis:** Added extensive discussion of why U=U absence is particularly concerning: incorporated into Ghana guidelines in 2019 (5 years before study), internationally recognized since 2016, yet 0% of our participants aware despite many having sustained viral suppression. This represents more fundamental counseling failure than absence of knowledge about more complex/expensive technologies like IVF. **(4) New thematic section:** Added dedicated subsection in Theme 1 results specifically on "Missing Information on U=U for Safer Conception" with participant quotes showing continued fear despite undetectable viral loads. | **Results - Table 2:** Page 16, U=U row shows 0% awareness; **Results - Table 4:** Page 20, U=U repositioned as first row (most basic/essential information); **Results - Theme 1:** Pages 17-18, new subsection on U=U absence; **Discussion:** Pages 23-24, extensive discussion of U=U absence implications |
| **ADDITIONAL SUGGESTION: Maternal Mortality in Background, Discussion, Conclusion** | "Suggest adding to the background, as well as the discussion and conclusion section, the importance and impact proper reproductive health access to women living with HIV would have on maternal mortality and vertical transmission of HIV, considering the importance those topics have in the region." | Addressed comprehensively as described in Weakness #3 response above. We have integrated maternal mortality and vertical transmission throughout Introduction, Discussion (new major subsection), and Conclusion with 15 new supporting references from WHO, UNAIDS, and Ghana-specific studies. | As detailed in Weakness #3 response above |
